# Strategic risk analysis for the selection of stable and high-potential maize genotypes in multi-environment trials

**DOI:** 10.1371/journal.pone.0325454

**Published:** 2025-06-06

**Authors:** Mohammadreza Shiri, Sajjad Moharramnejad, Afshar Estakhr, Sharareh Fareghi, Hamid Najafinezhad, Saeed Khavari Khorasani, Aziz Afarinesh, Morteza Eshraghi-Nejad

**Affiliations:** 1 Department of Maize and Forage Crops Research, Seed and Plant Improvement Institute, Agricultural Research Education and Extension Organization (AREEO), Karj, Iran; 2 Crop and Horticultural Science Research Department, Ardabil Agricultural and Natural Resources Research and Education Center, AREEO, Moghan, Iran; 3 Crop and Horticultural Science Research Department, Fars Agricultural and Natural Resources Research and Education Center, AREEO, Shiraz, Iran; 4 Crop and Horticultural Science Research Department, Kermanshah Agricultural and Natural Resources Research and Education Center, AREEO, Kermanshah, Iran; 5 Crop and Horticultural Science Research Department, Kerman Agricultural and Natural Resources Research and Education Center, AREEO, Kerman, Iran; 6 Crop and Horticultural Science Research Department, Khorasan Razavi Agricultural and Natural Resources Research and Education Center, AREEO, Mashhad, Iran; 7 Crop and Horticultural Science Research Department, Safiabad Agricultural and Natural Resources Research and Education Center, AREEO, Dezful, Iran; 8 Crop and Horticultural Science Research Department, South Kerman Agricultural and Natural Resources Research and Education Center, AREEO, Jiroft, Iran; ICAR - Indian Agricultural Research Institute, INDIA

## Abstract

Plant breeders are increasingly utilizing stability parameters as valuable tools for selecting cultivars in the context of genotype × environment interaction (GEI). Neglecting GEI in multi-environment trials (MET) can significantly heighten the risk of making inaccurate cultivar recommendations to farmers. Consequently, breeders must strive to find an optimal balance between yield and stability, favoring varieties that minimize the risk of extremely low yields. Recent advancements in probability theory, along with specialized software packages, have made the decision-making process more efficient for identifying suitable candidates across diverse environments. Under this scenario, a study was conducted to evaluate 15 promising maize hybrids alongside one control commercial hybrid (hybrid No. 16). The research employed a randomized complete block design with four replications across eight diverse locations over two consecutive years. The hybrids evaluated resulted from crosses involving temperate × temperate and tropical/subtropical × temperate. The objectives of this research included estimating the stability of these hybrids and assessing the associated risks related to their release, as well as evaluating the success and potential of lines derived from subtropical and tropical materials. A Bayesian approach was applied to estimate the probability that each genotype outperformed its competitors. The variance component estimates indicated that **“**location**”** was the most significant factor influencing overall variability, with values of 0.756 for genotype, 11.304 for location, and 0.621 for genotype × location effects. To enhance the mean grain yield within the selection panel, a selection intensity of 20% was implemented based on computed probabilities of superior performance and stability among selected candidates. Hybrid H2 exhibited the highest probability of superior performance (0.99), closely followed by Hybrid H5 with 0.97 of probability of belonging to the top subset. Hybrid H2 outperformed hybrid H16 (check hybrid) in all cases across tested environments; however, it demonstrated lower stability in 55% of comparisons. This finding suggests that the hypothesis asserting H2’s superiority over H16 in both stability and performance was not supported. H5 was the only hybrids common to both the top-performing (H2, H5, H4, H3) and most stable (H13, H15, H5, H7) groups. It is essential for breeders to jointly consider the probabilities of superior performance and stability when determining optimal genotypes. Considering the joint probability of superior performance and yield stability, the hybrids H5, H4, H15 and H2 stand out. High-performing and stable hybrids like H5 and H2 reduce cultivar introduction risks. Overall, these results indicated that employing a risk/probability analysis approach can significantly enhance decision-making accuracy for cultivar recommendations in METs.

## Introduction

Assessing crops in trials across diverse environments is essential for crop improvement, as it helps establish consistency and informs data-driven decisions regarding crop performance. The varying responses of different genotypes to these environments, known as genotype-environment interaction (GEI), represent one of the most significant challenges in crop improvement. Multi-environment trials (MET) are conducted to analyze the effects of GEI on crop performance and to develop cultivar recommendations tailored to target areas [1,2]. Typically, the relative merit of different cultivars is determined not only by their average yield but also by the stability of that yield. Today, cultivar recommendations also incorporate stability analysis methods, allowing for more reliable recommendations that consider both yield and yield stability [3]. Numerous yield-based stability methods including univariate and multivariate approaches that plant breeders can use to determine the best cultivars when GE interactions are present. Univariate methods, such as the Eberhart-Russell regression model, Shukla’s stability variance, and Finlay-Wilkinson regression model, provide valuable information that helps breeders rank or classify the adaptability of cultivars [3]. However, these methods do not offer a clear framework for developing a usable index that integrates both mean yield and stability. With that, further sophisticated methods have been employed such as the principal component analysis (PCA) of residuals from analysis of variance (ANOVA) tables and genotype and genotype by environment interaction (GGE Biplot) analysis of genotype and genotype-environment interactions [4], site regression model [3], and factor analytic multiplicative mixed model [5] have become widely used. In recent times, there has been an increased interest in the analysis of yield stability, given that, indeed, climate variability has been associated with lower crop yield stability [6,7]. Farmers are interested in temporal yield stability since it affects economic predictability, and also minimizes the risk [3]. Plant breeders accept latent risks when selecting and recommending experimental genotypes suited to a particular environment. Although multivariate stability methods prioritize the simultaneous selection of stability and mean yield, they have limitations that must be considered. These methods do not take into account the measurement of uncertainty in the phenotypic response to environmental gradients, which is a critical factor for improving crop compatibility and providing accurate cultivar recommendations.

Considering the GEI, any yield prediction from the statistical analysis of METs is subject to uncertainty. Specifically, measuring or quantifying the risk associated with performance is particularly important in breeding programs. Performance risk refers to the uncertainty related to the yield of a specific cultivar in a given environment, which arises from the GEI. To manage performance risk, it is useful to quantify the probability that a particular cultivar will perform better than others in a defined set of environments. This can be accomplished by calculating the probability of a cultivar outperforming other cultivars within a specific group [8] or by estimating the probability (risk) that a cultivar’s yield falls below a certain threshold [9–11].

Utilizing probability-based approaches can be one of the critical success factors to enhance our skill in identifying consistent cultivars across different environments or breeding regions. A great number of research works have used the frequentist approach to quantify risk and genotype ranking in MET [3,12,13]. Additionally, the Bayesian approach has proven to be a valuable method for incorporating prior information from previous experiments [2,5,6,14], analyzing GEI [5,12,15], and stability variances [7,15]. These advantages help in understanding GEI mechanisms and predicting the performance of new genotypes in untested environments.

Recently, Dias et al. [14] introduced a novel Bayesian approach that incorporates risk defined as “the potential for loss or underperformance” during the selection process. The posterior distribution plays a key role in the determination of yield and stability probabilities using Hamiltonian Monte Carlo. Their approach contains two elements: the probability of being selected as an indicator of the performance of an experimental genotype in a transect and the probability that a genotype that is selected performs consistently across environments. The pairwise probabilities produced in this manner are useful for comparing the performance of one experimental genotype with another or with standard cultivars.

Any efficient statistical method requires implementation, for which a proper suitable computer program is necessary to visually present the results. In this regard, a new user-friendly package (ProbBreed package) has been developed in the R environment, enabling users to assess cultivar recommendation risk and visualize data from multi-environment trials (MET) using probability theory under a Bayesian framework [14]. This package allows breeders to assess the probability that a particular genotype will surpass competitors by employing a Bayesian methodology. It simulates a real situation commonly faced by growers: the selection of the cultivar(s) that is more likely to be in optimal condition during the next cropping season. In addition, according to the intensity of the selection, probabilities-marginal or conditional-are computed; this is also part of plant breeders’ routine. Finally, in contrast with the biplot-based methods [4], selection is straightforward since there is only one metric to be used for selection, that is, the computed probabilities.

Two commonly used and effective multivariate models for analyzing stability and yield trials, in plant breeding programs, are additive main effects and multiplicative interaction (AMMI) and GGE Biplot. These models are used to assess the adaptability and stability of genotypes and to identify mega environments [16–18]. The AMMI model aids in identifying high-yield genotypes that are well-suited to a specific agronomic zone, focusing on the assessment of environmental influences. AMMI and GGE biplot analyses combine PCA and a graphical explanation of GEI. The AMMI model integrates ANOVA and PCA as an efficient procedure for an analysis of the stability of genotypes in a MET [18]. Despite progress in studies focused on understanding and utilizing genotype-environment interaction (GEI) through adaptation and stability, plant breeding still needs clearer methodologies to ensure more reliable variety recommendations [19]. In this context, the approach proposed by Dias et al. [14] stands out as a promising method for enhancing decision-making in variety recommendations. Consequently, the purpose of this study was to investigate the application of a Bayesian probabilistic method to analyze GEI, select high-performing and stable maize hybrids, and evaluate the risks associated with maize variety recommendations.

## Materials and methods

### Experimental materials and design

A set of 15 promising maize hybrids, along with one commercial check (hybrid No. 16) (S1 Table) were allocated in a randomized block design across eight different locations: Karaj (35.49N, 5100E), Shiraz (25.46N, 52.43E), Kermanshah (34.80N, 47.26E), Mashhad (32.59N, 36.19E), Moghan (39.41N, 47.32E), Kerman (28.45N, 56.36E), Jiroft (28.40N, 57.44E), and Dezful (32.75N, 48.20E) during two growing seasons (2022–2023). In the second year of the experiment, however, outbreaks of fall armyworm (*Spodoptera frugiperda*) in the Kerman and Jiroft regions caused substantial damage to the trials, leading to the removal of these two locations from the study.

The experimental locations were situated at altitudes ranging from 73 to 1600 m above sea level, with latitude ranging from 25.46 to 57.44 in Iran (S2 Table). Each plot consisted of four 6.5 m rows spaced 0.75 m apart, with 0.36 m between hills containing two plants each. The planting density was approximately 74,000 plants/ha. To ensure optimal seed emergence, three seeds were planted at each hill, and thinning was conducted at the 4–5 leaf stage (approximately 18 days after planting) to retain only two plants per hill. All recommended agronomic practices, including irrigation, weed control, and fertilization, were implemented to promote good crop growth across all locations. The amount of fertilizer was determined based on soil tests. Grain yield was then measured at the moisture content of 14% [20]. The hybrids studied were developed by crossing lines from temperate regions with each other, as well as by crossing tropical and subtropical lines with temperate lines.

### Statistical analysis

#### Model description for Bayesian MET approach.

We employed the probabilistic approach outlined by Dias et al. [14], implementing two Bayesian models based on two assumptions: homogeneous residual variance and heterogeneous (diagonal) residual variance. We fitted four chains, each consisting of 2000 samples. To improve the quality of our results, we discarded 50% of the initial samples as burn-in, resulting in a total of 4000 samples. The posterior probability distributions of the model parameters were derived using the probabilistic programming language Stan [21,22] through the RStan interface [23]. Utilizing Bayesian models, including the No-U-turn Sampler and Hamiltonian Monte Carlo algorithm, has demonstrated enhancements in computational efficiency while also removing the requirement to determine the number of leapfrog updates. This helps prevent random-walk behavior and boosts overall computational effectiveness [21,22].

The initial model was based on the assumption of uniform residual variance and was defined by the subsequent conditional normal probability [14]:


$$y_{ijk}\sim N(E[y_{ijk}],\sigma )$$

$$E[y_{ijk}]=\mu +g_{i}+r_{j}+e_{k}+{ge}_{ik}+p_{ij}+e.$$

Here, $E[y_{ijk}]$ represents the expected phenotype for the *i*^*th*^ genotype evaluated in the *j*^*th*^ block at the *k*^*th*^ environment; *σ* is the standard deviation; *μ* is the overall mean; *g*_*i*_ denotes the genotypic effect; *r*_*j*_ indicates the block effect; *e*_*k*_ is the environment effect; *ge*_*ik*_ represents the genotype-by-environment interaction; *p*_*ij*_ accounts for the environmental permanent effect; and *e* is the residual error. The prior probability distribution for each model parameter was defined as follows [14]:


$$\mu \sim N\left(0,\;\sigma_{\left[\mu \right]}\right);$$

$$r\sim N\left(0,\sigma_{\left[r\right]}\right);$$

$$e\sim N\left(0,\sigma_{\left[e\right]}\right);$$

$$g\sim N\left(0,\sigma_{\left[g\right]}\right);$$

$$ge\sim N\left(0,\sigma_{\left[\mu \right]}\right);$$

$$p\sim N\left(0,\sigma_{\left[p\right]}\right);$$

$$e\sim Half\;Cauchy(0,\sigma_{\left[e\right]});$$

$$a\sim Half\;Cauchy(0,\sigma_{\left[a\right]})$$

In this context, $N(0,\sigma_{\left[\alpha \right]})$ and $Half\;Cauchy(0,\sigma_{\left[\alpha \right]})$ denote the normal and half-Cauchy distributions, respectively, with a mean of zero and varying scale parameters

$\sigma_{\left[\alpha \right]}^{2}$. The following hyper priors were applied for the respective parameters:


$$\sigma_{\left[\mu \right]}\sim Half\;Cauchy(0,\phi );$$

$$\sigma_{\left[r\right]}\sim Half\;Cauchy(0,\phi );$$

$$\sigma_{\left[e\right]}\sim Half\;Cauchy(0,\phi );$$

$$\sigma_{\left[g\right]}\sim Half\;Cauchy(0,\phi );$$

$$\sigma_{\left[ge\right]}\sim Half\;Cauchy(0,\phi )$$

Where *ϕ* is a predetermined global hyperparameter (ϕ=max(y)×10), ensuring weakly informative second-level hyperpriors. Consequently, the data predominated the posterior distributions. The half-Cauchy distribution is typically recommended for modeling variance parameters due to its restriction to positive values.

The second Bayesian model incorporated heterogeneous (diagonal) residual variances. This model maintained the same assumptions as the first model but allowed for $\sigma_{k}HalfCauchy(0,\sigma_{\left[\sigma k\right]})$.

#### Model selection and convergence diagnostics.

The scale reduction factor ($\hat{R}$) was utilized to evaluate how effectively the Markov chain Monte Carlo (MCMC) converged, measuring the variation both within and between chains, indicating the degree of equilibrium among the chains for the model parameters. Actually, it is the ratio between the average variance of samples within chains and the variance among chains. Thus, values closer to one imply these variances are similar, indicating good convergence among samples from different chains within the parameter space, which is desirable [24]. Conversely, an R̂ value greater than one suggests poor mixing of the Markov chains in the Bayesian analysis. Higher R̂ (greater than one) values indicate that more iterations are needed to achieve convergence and reliable parameter estimates [25]. Additionally, the WAIC2 (Watanabe-Akaike Information Criterion) was utilized to compare models and estimate their out-of-sample predictive accuracy, representing a fully Bayesian approach to model selection and evaluation of predictive performance. The log pointwise posterior predictive density is a metric in Bayesian statistics that assesses how well a model predicts new data based on observed data. It is defined as the logarithm of the probability of observing new data points, given the existing data and the model parameters. WAIC2 provides a robust framework for assessing the predictive capabilities of different models by estimating the log pointwise posterior predictive density. WAIC2 allows researchers to quantify the trade-off between model fit and complexity, thereby facilitating the selection of models that not only explain the observed data well but also generalize effectively to generated data.

Graphical analysis first considers the fitness of the data generated by our model to the true generative process of the observed data. This may be done by performing ancestral sampling from the conditional joint distribution of fitted models and computing samples, (“$y_{gen}$”), plotting those against the observed data. Moreover, we also utilized posterior predictive *p*-values in an attempt to examine if the distributional statistics-mean, standard deviation, median, maximum, and minimum-of data simulated from the fitted models are close to the observed data [25]. For instance, for the mean statistic, the Bayesian *p*-value was defined as:


$$P_{mean}=pr(y_{gen},\theta )\geq T(y,\theta )|y$$

Where T represents the test statistic. As the Bayesian p-values approach 0.5, the degree of similarity between the statistics derived from the generated data and those from the observed data increases, indicating a better fit of the model to the actual breeding data.

#### Probability of superior performance.

Consider a dataset in which *J* genotypes (*j* = 1, 2,..., *J*) were evaluated at *K* environments (k = 1, 2,..., K) with *y* observed phenotypes. Let Ω be a subset of the high-performance selected genotypes according to the intensity of selection (3 out of 16 corresponding to 20% selection intensity).

We consider that a genotype *j* belongs to Ω if its respective genotypic marginal value, (${\hat{g}}_{j}$) is sufficiently high or low compared to other genotypes. For Bayesian models, we simulated *S* trials, indexed by (s = 1, 2,..., S) via Monte Carlo samples from the posterior distributions. We calculate the probability of genotype j belonging to Ω, as a ratio of successful events where ${\hat{g}}_{j}\in \Omega$ over total number of sampled events defined as (${S=(\hat{g}}_{j}\in \Omega )+{(\hat{g}}_{j}\notin \Omega )$) [14].


$${Pr(\hat{g}}_{j}\in \Omega |y)=\frac{1}{S}\sum_{1}^{S}I({\hat{g}}_{j}\in \Omega |y)$$

Where $I({\hat{g}}_{j}^{\left(s\right)}\in \Omega_{k}|y)$ is an indicator variable that can assume two values: if

${\hat{g}}_{j}\in \Omega$ in the $s^{th}$ sample, and (0) otherwise.

Similarly, the conditional probability of superior performance can be applied to individual environments. Let $\Omega_{k}$ represent the subset of superior genotypes in the kth environment, so that the probability of the${\;j}^{th}\in \Omega_{k}$ can calculated as follows:


$$Pr({\hat{g}}_{jk}^{\left(s\right)}\in \Omega_{k}|y)=\frac{1}{S}\sum_{1}^{S}I({\hat{g}}_{jk}^{\left(s\right)}\in \Omega_{k}|y)$$

Where I$\left({\hat{g}}_{jk}^{\left(s\right)}\in \Omega_{k}|y\right)$ is an indicator variable mapping success if ${\hat{g}}_{jk}^{\left(s\right)}$exists in $\Omega_{k}$, failure otherwise, and ${\hat{g}}_{jk}^{\left(s\right)}={\hat{g}}_{j}^{\left(s\right)}+{\hat{ge}}_{jk}^{\left(s\right)}$. Note that computing conditional probabilities (i.e., conditional to the $k^{th}$ environment or mega-environment), the interaction of the $j^{th}$ genotype with the $k^{th}$ environment is accounted [14].

The pairwise probabilities of superior performance can also be calculated across or within environments. This metric assesses the probability of the $j^{th}$ genotype being superior to another experimental genotype or a commercial check. The calculations are as follows [14]:


$$Pr({\hat{g}}_{j}>|y)=\frac{1}{S}\sum_{1}^{S}I({\hat{g}}_{j}^{\left(s\right)}>{\hat{g}}_{j^{\prime }}^{\left(s\right)}|y)$$

$$Pr({\hat{g}}_{jk}>{\hat{g}}_{j^{\prime }k}|y)=\frac{1}{S}\sum_{S=1}^{S}I({\hat{g}}_{jk}^{\left(s\right)}>{\hat{g}}_{j^{\prime }k}^{\left(s\right)}|y)$$

#### Probability of superior stability.

The probability of attaining high performance indicates the presence of high-performance genetic traits. For stability, it is more appropriate to consider the probability of achieving superior stability. This measure can be compared directly to Zhou et al. [26] method that defines a stable genotype as having low variance in the effects of GEI [$var(\hat{ge)}$]. By applying the same concept of probabilities as discussed above, the probability of superior stability is determined as follows [14]:


$${Pr[var(\hat{ge}}_{jk})\in \Omega |y]=\frac{1}{S}\sum_{S=1}^{S}I[var({\hat{ge}}_{jk}^{\left(s\right)}>\in \Omega |y)$$

Where $I[var({\hat{g}}_{jk}^{\left(s\right)})\in \Omega |y]$ indicates if $var({\hat{ge}}_{jk}^{\left(s\right)}$ exists in Ω. Note that this probability can only be computed across environments since it depends on ${var(\hat{ge}}_{jk})$. Pairwise probabilities of superior stability are also computed in the context of stability [14]:


$${Pr[var(\hat{ge}}_{jk})<{var(\hat{ge}}_{j^{\prime }k})|y]=\frac{1}{S}\sum_{S=1}^{S}I[(var{\hat{ge}}_{jk}{)}^{\left(s\right)}<var{\hat{ge}}_{ik}{)}^{\left(s\right)}|y)$$

#### Joint probability of superior performance and stability.

Since independent events occur in a probability joint with a product of individual probabilities, and as the estimated genotypic main effects and variances of GEI effects are independent by design through linear models, the joint probability of superior performance and stability is given as follows [14]:


$${Pr[\hat{g}}_{j}\in \Omega \cap {var(\hat{ge}}_{jk})\in \Omega ]={Pr(\hat{g}}_{j}\in \Omega )\times {Pr[var(\hat{ge}}_{jk})\in \Omega ]$$

## Results

### Model description for Bayesian MET approach

To identify the best-fitting Bayesian model based on two assumptions, homogeneous residual variance, and heterogeneous (diagonal) residual variance, it was found that both models exhibited potential scale reduction factors (R̂) close to one; however, the R̂ for the heterogeneous (diagonal) residual variance model was exactly one, indicating strong convergence of the model parameters. Furthermore, the Bayesian model with heterogeneous (diagonal) residual variance showed a lower WAIC2 value, suggesting it provides a better fit for the data compared to the homogeneous residual variance model. The ability of the heterogeneous (diagonal) residual variance model to achieve a perfect fit demonstrates its effectiveness in accommodating the complexities inherent in the data, thereby offering a more reliable foundation for subsequent estimates and enhancing predictive accuracy.

**Fig 1:**
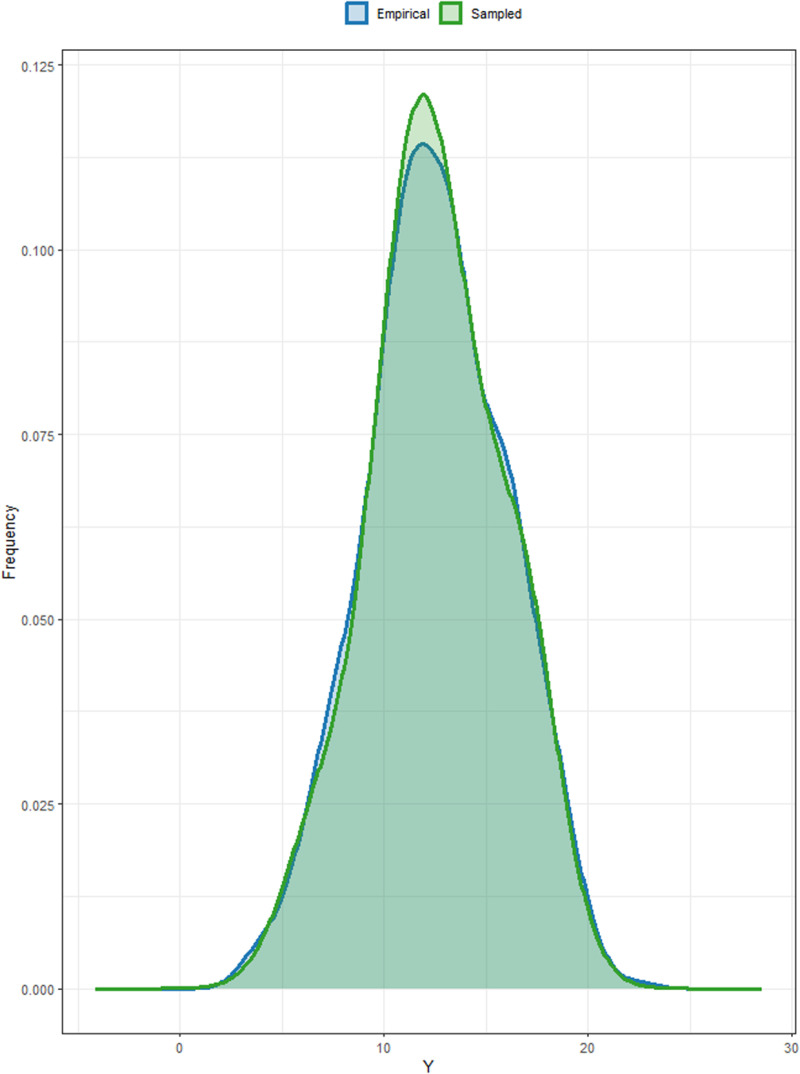
Density plot of the data generated in comparison to the distribution of the real data.

We then used ancestral sampling from the conditional joint distribution to generate samples from the fitted models and assessed well the model-produced data represented the actual generation process by comparing these results with the observed data through plotting. The comparison of the data produced by the Bayesian model with heterogeneous (diagonal) residual variance revealed that the density produced by the model closely follows the trend of the observed data. This highlights the model’s effectiveness in replicating the distribution of the observed data, emphasizing its reliability in capturing the underlying patterns (Fig 1).

### Posterior effects and goodness-of-fit diagnostics

The Bayesian “*p*-values” were computed concerning the probability of test statistic maximum, minimum, median, mean, and standard deviation in generated data being higher than in real data are computed. If the generated data is a good match for the observed data, Bayesian p-values should not lie at the tails, that is, 0.99 or 0.01. In principle, a Bayesian p-value of 0.5 is preferred as it was indicative of a better resemblance between the statistics of generated and observed data. Comparing test statistics from the observed data with those from generated data yielded Bayesian *p*-values of between 0.1779 and 0.6479 (Table 1). These results showed that the data statistics were not in a significant deviation from those obtained from the observed data.

**Table 1:** Goodness-of-fit parameters: Bayesian “*p*-values” of test statistics [maximum, minimum, median, mean, and standard deviation], the effective number of parameters, potential scale reduction factor ($\hat{R}$), and effective sample size.

Parameter	Homogeneous residual variance	Heterogeneous (diagonal) residual variance
*p*-value of the maximum	0.1974	0.1779
*p*-value of the minimum	0.2385	0.1966
*p*-value of the median	0.7148	0.6479
*p*-value of the mean	0.4925	0.5089
*p*-value of the std. deviation	0.5212	0.6156
Effective number of parameters	77.0943	86.9409
$\hat{R}$	0.9999	1.0002
Effective sample size	1.4118	1.0763
WAIC2	3865.6688	3794.5258

### Bayesian variance components estimate for MET data

The estimated variance components for the genotype (G), location (L), and their interaction (G × L) effects were 0.756 for the genotype, 11.304 for location, and 0.621 for the G × L interaction (Table 2). These estimates provide valuable insights into the variability associated with each effect in the Bayesian model. Notably, the “Location” effect exhibits the highest variability, as evidenced by its significantly larger variance component compared to the others. This indicates that the “Location” factor plays a crucial role in contributing to the overall variability in the model.

**Table 2:** Bayesian estimates of variance components for the declared effects, along with their respective standard deviation (sd), naive standard error (Naive.se), and inferior and superior high posterior density interval [HPD (0.05) and HPD (0.95)].

Effect	Variance	sd	Naive.se	HPD_0.05	HPD_0.95
Replication	0.292	0.1	0.001	0.154	0.475
Genotype(G)	0.756	0.379	0.004	0.342	1.455
Location(L)	11.304	5.616	0.063	5.279	21.901
G × L	0.621	0.118	0.001	0.44	0.829
Residual variance for 14 environments					
Env1	3.349	0.668	0.007	2.393	4.563
Env2	2.226	0.465	0.005	1.583	3.077
Env3	3.827	0.754	0.008	2.787	5.206
Env4	2.003	0.408	0.005	1.428	2.74
Env5	1.562	0.338	0.004	1.091	2.176
Env6	1.681	0.35	0.004	1.193	2.328
Env7	1.286	0.276	0.003	0.892	1.788
Env8	5.255	1.051	0.012	3.747	7.172
Env9	2.78	0.573	0.006	1.972	3.818
Env10	1.322	0.284	0.003	0.925	1.846
Env11	4.773	0.996	0.011	3.376	6.652
Env12	1.62	0.37	0.004	1.127	2.287
Env13	1.883	0.428	0.005	1.305	2.671
Env14	1.62	0.355	0.004	1.124	2.274

### Probabilities of superior performance and genotypic stability of hybrids

**Fig 2:**
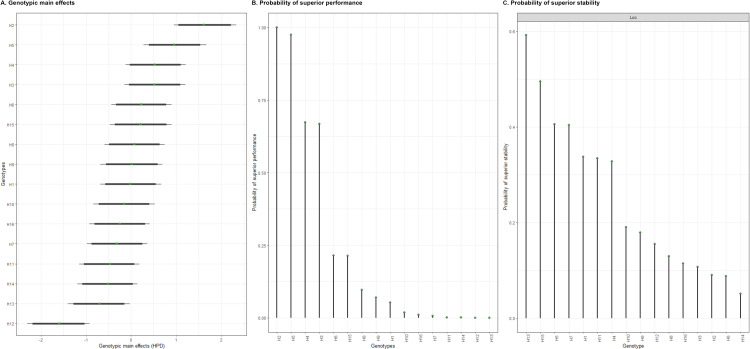
Highest posterior density (HPD) of the posterior genotypic main effects (A), probability of superior performance across environments (B), and probability of superior stability across locations (C). In (A), the dots represent the maximum posterior values, while the thick and thin lines indicate the 95% and 97.5% HPD intervals, respectively. The x-axes of (B) and (C) are sorted in decreasing order based on the computed probabilities.

To evaluate the improved performance and stability probabilities among the selection candidates, we used a selection intensity of 20% to attain an upward shift in the average grain yield for the selected hybrid panel. The performance of the selection candidates for different environments is summarized by a caterpillar plot displaying the posterior genotypic main effects along with their respective HPD (Highest posterior density) intervals (Fig 2). Therefore, the posterior distribution of the genetic performance for maize hybrids (gj) came to a different pattern of their HDI overlap. Among them, hybrids H2 and H5 had the highest genetic performance, while hybrids H12 and H13 showed the lowest genetic performance (Fig 2A). It is relevant to mention that the maximum posterior values correspond to marginal empirical best linear unbiased predictions - BLUPs - obtained under a frequentist specification via linear mixed models, considering independent genotypic effects. Since the probability of performance was calculated based on the estimated genetic performance, it was anticipated that the ranking of the top-performing hybrids would align with the results derived from the highest posterior density interval (HDI) values (Fig 2 A and B). Hybrids H2, H5, H4, and H3 revealed the greatest probabilities of superior performance (Fig 2B). Hybrids H11, H14, H12, and H13 were not selected in any of the samples, while the remaining 8 hybrids were selected in at least some samples (Fig 2B). Notably, Hybrids H2 and H5 were the candidates with the highest probability of superior performance, estimated at approximately 99.9% and 97%, respectively (Fig 2B). This indicates a 0.1% and 3% risk of poor performance, contingent upon selection intensity.

The probability of superior stability analysis suggested that across locations, hybrids H13, H15, H5, and H7 had the highest probabilities of superior stability, meaning that they have less variable performance in different environments (Fig 2C). Overall, the probability of identifying a maize hybrid with minimal variation in stability was relatively low in this study. Notably, only hybrid H13 exhibited the highest stability value, with an above 50% chance of exhibiting superior stability, indicating it to be the most stable hybrid among those evaluated (Fig 2C).

The analysis underscores the impact of different probability metrics on the results, emphasizing the necessity of establishing clear objective criteria before conducting analyses. If performance is the primary focus, Fig 2B should be referenced; conversely, if stability is the ultimate goal, priority should be given to Fig 2C.

**Fig 3:**
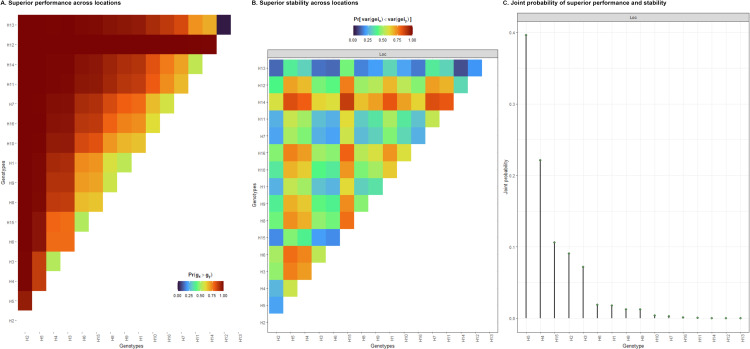
Pairwise probabilities of superior performance across locations (A), superior stability across locations (B), and joint probability of superior performance and stability (C). The heatmaps in (A), and (B) illustrated the probability of genotypes on the x-axis being superior to those on the y-axis.

Besides the individual probabilities, pairwise probabilities were also calculated to compare genotypes (Fig 3A and B). This analysis allows for estimating the chances that a hybrid will be among the best ones with respect to selection intensity. A heatmap showing all pairwise probabilities of superior performance were employed to compare the genetic performance of hybrids (Fig 3A). Hybrids H2 and H5 were invariably the best-performing hybrids, while H13 was invariably among those expressing the poorest relative performance. In comparison with the commercial check H16, H2 out-yielded H16 in all cases across tested environments while being less stable in 55% of the cases. H5 out-yielded H16 in 99% of cases while being highly stable in 75% of the cases (Fig 3A and B). If breeders are interested in genotypes with superior performance and stability, they would look to Fig 3C showing the joint probability of superior performance and stability. Although it is very unlikely that a single genotype will stand out in all probability metrics, by both performance and stability criteria, hybrid H5 is considered the most promising candidate.

**Fig 4:**
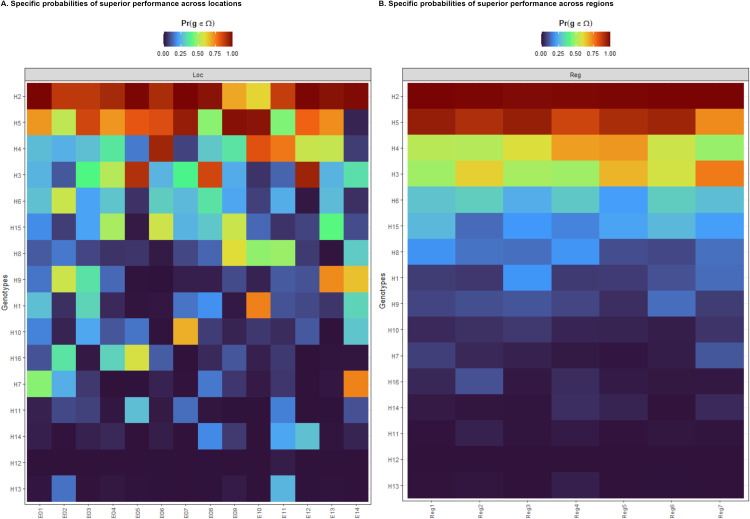
Heatmaps representing the specific probabilities of superior performance across locations (A) and regions (B). The following locations are represented: E01, E09: Karaj; E02: Kerman; E03, E10: Mashhad; E04, E11: Kermanshah; E06, E12: Moghan; E05: Jiroft; E07, E13: Shiraz; E08, E14: Dezful. The following regions are represented: E01, E09: Reg1; E02, E05: Reg2; E03, E10: Reg3; E04, E11: Reg4; E06, E12: Reg5; E07, E13: Reg6; E08, E14: Reg7.

In plant breeding, it is crucial to identify the locations where a hybrid is likely to excel or underperform. Understanding the probability of superior performance in different environments assists breeders in making informed decisions for both broad and specific variety recommendations (Fig 4A). For instance, hybrid H2, recognized as the top performer in various environments, may underperform at locations E9 and E10; however, it has a greater chance of success in other locations. If E9 and E10 are important for maize production, it may be advisable to recommend hybrid H5 for those locations. In addition to evaluating the success or failure of a variety’s release in a specific location, this recommendation can also be extended to a broader region, which may include a combination of years and locations or sets of similar environments (Fig 4B). In this context, it was anticipated that introducing the promising hybrid H2 would have a higher probability of success compared to the promising hybrid H5.

## Discussion

The consideration of genotype by GEI is critical in the genetic evaluation of crops, as gene expression varies in response to different environmental factors [27]. In plant breeding programs over the past two decades, statistical models such AMMI and GGE Biplot have been widely used to evaluate performance and stability. Both statistical methods are based on PCA that provides a particular way to assess the relationships between environments, genotypes, and genotypes with environments. Moreover, both methods can display a graphical view of the results, which in turn results in a better understanding of the response of test genotypes in different environments [18]. The literature provides many examples of the AMMI model application in the studies of GEI shape, for various features of high economic importance species, such as maize [28], wheat [16], sugar beet [17], or *Plantago* [18]. The Bayesian probabilistic method streamlines traditional techniques for investigating GEI, offering fresh insights into phenotypic plasticity through probabilistic approaches. Previous studies by Filipe and Kyriazakis [29], later modified by Brooker et al. [30], Arata et al. [6], and Chaves et al. [12] employed probability-based methods along with additional options to assist breeders in cultivar selection and recommendation. The proposed models fully utilize the posterior distribution obtained with the No-U-Turn sampler algorithm [24] to calculate posterior probabilities, allowing for straightforward consideration of the uncertainty associated with each parameter. With the properties of Markov Chain Monte Carlo (MCMC), theoretically, when using the exact unknown cumulative posterior probability distribution from non-conjugate Bayesian models with an asymptotically increasing sample size from the MCMC chain, the probabilities converge to the exact value [31–33]. Dias et al. [14] explored theoretical and practical concepts of applying probability methods to minimize decision-making risk in breeding programs, finding good agreement between the Bayesian approach and classical GEI models. To exploit the mentioned advantages, a Bayesian probabilistic approach was employed to understand GEI for making cultivar recommendations across different environments. We analyzed a maize dataset with phenotypic records of 16 single-cross hybrids evaluated in 14 environments in Iran.

The best hybrids, H2 and H5, have been to emerge as strong candidates for recommendation due to their highest probability of superior performance. These genotypes possess the necessary alleles for adaptability to different environmental conditions that will enable them to perform sufficiently well using probabilistic methods. Analysis of stability probabilities indicated that H13, H15, H5, and H7 exhibited the highest probabilities of superior stability, demonstrating less variability in performance across locations. Our findings are consistent with those of Malikouski et al. [33] and Miranda et al. [34], who also utilized Bayesian models to recommend cultivars. Their studies reinforced the effectiveness of these models in accurately estimating genotypic values and performance traits, enhancing breeding selection strategies.

One of the bottlenecks in METs is establishing reliable criteria to compare the risks associated with different cultivars. Traditionally, the performance of a check cultivar has served as the benchmark for such comparisons [7,35,36]. However, the inconsistency of check cultivar performance across various environments can lead to misleading conclusions about cultivar suitability and risk assessment [2,3,6,13,21,30]. To address this inconsistency, Lin and Binns proposed an index based on the distance to the maximum genotype response within each environment. This approach offers a more standardized method for evaluating cultivar performance by focusing on relative performance rather than absolute measures. Joint probabilistic models further enhance this approach by offering a comprehensive framework for cultivar adaptation, considering both global and specific adaptation. These models facilitate a nuanced understanding of cultivar performance, incorporating yield potential and stability across environments. By utilizing joint probabilities, breeders can better interpret the likelihood that a cultivar ranks among the superior options based on predefined selection intensities. The Bayesian safety-first index provides a probabilistic evaluation of genotype performance, taking into consideration both yield potential and stability across different environments. Genotypes with high values in this index are preferred, as they indicate a combination of high yield and consistent performance, making them suitable for general or specific adaptation.

By simultaneously selecting for superior performance and stability through joint probability, the hybrids H5, H4, H15, and H2 were recognized as the top-performing hybrids. The pursuit of methodologies to select high-yielding and stable genotypes across environments has been a longstanding challenge. In this context, Kang [37] proposed a rank-sum method, Eskridge [9] introduced a safety-first rule, and Annicchiarico [38] put forth a risk index that considers cultivar means and stability concurrently. Our results align with those of Malikouski et al. [33] and Miranda et al. [34], highlighting the effectiveness of Bayesian models in identifying superior genotypes.

Conducting all-pairwise comparisons among genotypes is essential for gaining a comprehensive understanding of their general comparative performance, particularly in advanced trials prior to cultivar release. This approach enables breeders to evaluate the relative strengths and weaknesses of each genotype, facilitating informed decision-making in the selection process. The probabilities computed using posterior samples account for parameter uncertainty, providing a more reliable assessment of genotype performance. This probabilistic framework enhances the breeding process by integrating genetic insights with environmental adaptability, ultimately leading to the development of more resilient and high-performing crop varieties. As noted earlier, based on the marginal probability of superior performance, hybrid H15 has been identified as the best-performing cultivar among the tested hybrids. This experimental hybrid is particularly promising, and further investigation is warranted to determine its performance relative to the commercial check, H16. Across various locations, H15 outperformed H16 98% of the time (Fig 3A). However, it is important to note that H15 exhibited less stable performance than H16 in 60% of the instances (Fig 3B). Consequently, there is insufficient evidence to support the hypothesis that hybrid H15 consistently outperforms the commercial check (H16) in terms of both stability and performance simultaneously. Our findings are consistent with the results of Malikouski et al. [33] on Tahiti acid limes and Miranda et al. [34] on common beans using the aforementioned methodology. These studies demonstrated the effectiveness of these models and reinforced their reliability for improving breeding selection strategies.

For breeders aiming to identify genotypes that excel in both high performance and stability, the analysis of joint probabilities is crucial. This analysis provides a comprehensive view of genotype performance, as illustrated in Fig 2C. It is essential to understand that it is unlikely for the same genotype to be the best across all probability metrics, highlighting the complexity of genotype evaluation in varying environmental conditions. The Bayesian model provides critical information on the uncertainty of a genotype belonging to the set of genotypes with specific or general adaptation. This information is invaluable in the selection process, allowing breeders to make informed decisions based on the probability of a genotype being among the top performers. Overall, the integration of these methodologies not only enhances our understanding of genotype performance but also paves the way for developing cultivars that are both high-yielding and stable across diverse environments.

The main question addressed in this research is the application of probability methods based on Bayesian models to improve the accuracy of cultivar recommendations for both global and specific adaptations. This is a crucial aspect of the decision-making process in any breeding program. The probability of specific adaptation provides valuable insights into genotype adaptation trends by allowing the identification of hybrids with desirable plasticity and performance tailored to specific breeding regions. This probabilistic approach enables breeders to make more informed decisions about cultivar suitability for targeted environments. Moreover, pairwise global adaptation analyses enhance the understanding of comparative performance among target genotypes across various environments. These analyses are often presented through user-friendly heatmaps, facilitating interpretation and decision-making. Finally, these findings provide a clearer and more comprehensive understanding of genotype-environment interactions based on a defined intensity of selection. By incorporating this information into their decision-making processes, breeders can make more informed cultivar recommendations during METs.

## Conclusion

Using probabilistic Bayesian models in the genetic evaluation, the probability of a genotype being superior was estimated; probabilities of superior performance between genotypes in different environments were estimated. Based on the results of various parameters-including marginal probabilities of superior performance (H2, H5, H4, and H3), marginal probabilities of superior stability (H13, H15, H5, and H7), and joint probabilities of superior performance and stability (H5, H4, H15, and H2)-hybrids H5 and H2 emerged as the top candidates for release as maize cultivars, offering an optimal balance of high yield and stability across diverse environments. Consequently, Bayesian probabilistic models can be of help in recommending the best maize crop that suits a particular region due to efficient evaluation of genotype performance and its persistence.

## Supporting information

S1 Table Basic information of the 38 tested maize hybrids. (DOCX)

Table S2 Description of the 14 environments with their respective latitudes, longitudes, altitudes (m), soil group, mean temperature, rainfall (mm), and climate condition in regard to the maize trial. (DOCX)

## References

[1] Haile GA, Tesfaye D. Response of field pea (Pisum sativum L.) genotypes for grain yield in a multi-environment trial in Southeastern Ethiopia. Heliyon. 2024;10(15). 10.1016/j.heliyon.2024.e35233 PMC11336437 39170294

[2] Rebollo I, Aguilar I, Pérez de Vida F, Molina F, Gutiérrez L, Rosas JE. Genotype by environment interaction characterization and its modeling with random regression to climatic variables in two rice breeding populations. Crop Science. 2023;63(4):2220–40.

[3] Reckling M, Ahrends H, Chen TW, Eugster W, Hadasch S, Knapp S. Methods of yield stability analysis in long-term field experiments. A review. Agronomy for Sustainable Development. 2021;41:1–28.

[4] Yan W, Hunt LA, Sheng Q, Szlavnics Z. Cultivar evaluation and mega‐environment investigation based on the GGE biplot. Crop Science. 2000;40(3):597–605.

[5] Oliveira ICM, Guilhen JHS, de Oliveira Ribeiro PC, Gezan SA, Schaffert RE, Simeone MLF. Genotype-by-environment interaction and yield stability analysis of biomass sorghum hybrids using factor analytic models and environmental covariates. Field Crops Research. 2020;257:107929.

[6] Arata L, Fabrizi E, Sckokai P. A worldwide analysis of trend in crop yields and yield variability: Evidence from FAO data. Economic Modelling. 2020;90:190–208.

[7] Hoff P. Additive and multiplicative effects network models. 2021.

[8] Piepho HP, van Eeuwijk FA. Stability analysis in crop performance evaluation. Crop Improvement. CRC Press; 2024. 315–51.

[9] Eskridge KM. Selection of stable cultivars using a safety‐first rule. Crop Science. 1990;30(2):369–74.

[10] Mead R, Riley J, Dear K, Singh SJB. Stability comparison of intercropping and monocropping systems. 1986;253–66.

[11] Piepho HP. A simplified procedure for comparing the stability of cropping systems. 1996;315–20.

[12] Chaves SF, Evangelista JS, Alves RS, Ferreira FM, Dias LA, Alves RM. Application of linear mixed models for multiple harvest/site trial analyses in perennial plant breeding. Tree Genetics & Genomes. 2022;18(6):44.

[13] Ruberg SJ, Beckers F, Hemmings R, Honig P, Irony T, LaVange L, et al. Application of Bayesian approaches in drug development: starting a virtuous cycle. Nat Rev Drug Discov. 2023;22(3):235–50. doi: 10.1038/s41573-023-00638-0 36792750 PMC9931171

[14] Dias KOG, Dos Santos JPR, Krause MD, Piepho H-P, Guimarães LJM, Pastina MM, et al. Leveraging probability concepts for cultivar recommendation in multi-environment trials. Theor Appl Genet. 2022;135(4):1385–99. doi: 10.1007/s00122-022-04041-y 35192008

[15] Lenartowicz T, Bujak H, Przystalski M, Piecuch K, Jończyk K, Feledyn-Szewczyk B. Yield stability and adaptability of spring barley (Hordeum vulgare) varieties in Polish organic field trials. Agronomy. 2024;14(9):1963.

[16] Ghaed-Rahimi L, Heidari B, Dadkhodaie A. Genotype× environment interactions for wheat grain yield and antioxidant changes in association with drought stress. Archives of Agronomy and Soil Science. 2015;61(2):153–71.

[17] Hassani M, Heidari B, Dadkhodaie A, Stevanato P. Genotype by environment interaction components underlying variations in root, sugar and white sugar yield in sugar beet (*Beta vulgaris* L.). Euphytica. 2018;214:1–21.

[18] Shahriari Z, Heidari B, Dadkhodaie A. Dissection of genotype × environment interactions for mucilage and seed yield in Plantago species: Application of AMMI and GGE biplot analyses. PLoS One. 2018;13(5):e0196095. doi: 10.1371/journal.pone.0196095 29715274 PMC5929508

[19] Smith AB, Cullis BR. Plant breeding selection tools built on factor analytic mixed models for multi-environment trial data. Euphytica. 2018;214:1–19.

[20] Rawal N, Vista SP, Khadka D, Paneru P. Grain yield, nitrogen accumulation, and its use efficiency of mize (*Zea mays* L.) as influenced by varying nitrogen rates. International Journal of Agronomy. 2024;2024(1):4104123.

[21] Carpenter B, Gelman A, Hoffman MD, Lee D, Goodrich B, Betancourt M, et al. Stan: A Probabilistic Programming Language. J Stat Softw. 2017;76:1. doi: 10.18637/jss.v076.i01 36568334 PMC9788645

[22] Williams B. Generalized no-u-turn sampler for Lagrangian Monte Carlo. University of Helsinki; 2022.

[23] Team RC. A language and environment for statistical computing. 2021.

[24] Fabreti LG, Höhna S. Convergence assessment for Bayesian phylogenetic analysis using MCMC simulation. Methods in Ecology and Evolution. 2022;13(1):77–90.

[25] Lye A, Cicirello A, Patelli E. An efficient and robust sampler for Bayesian inference: Transitional ensemble Markov chain Monte Carlo. Mechanical Systems and Signal Processing. 2022;167:108471.

[26] Zhou J, Gandomi AH, Chen F, Holzinger A. Evaluating the quality of machine learning explanations: A survey on methods and metrics. Electronics. 2021;10(5):593.

[27] Boyce WT, Sokolowski MB, Robinson GE. Genes and environments, development and time. Proc Natl Acad Sci U S A. 2020;117(38):23235–41. doi: 10.1073/pnas.2016710117 32967067 PMC7519332

[28] Shiri M, Moharramnejad S, Estakhr A, Fareghi S, Najafinezhad H, Khavari Khorasani K. Determining the stability of new maize hybrids with WAASBY and MTSI indices. Journal of Crop Breeding. 2024;16(2):14–28.

[29] Filipe JAN, Kyriazakis I. Bayesian, Likelihood-Free Modelling of Phenotypic Plasticity and Variability in Individuals and Populations. Front Genet. 2019;10:727. doi: 10.3389/fgene.2019.00727 31616460 PMC6764410

[30] Brooker R, Brown LK, George TS, Pakeman RJ, Palmer S, Ramsay L, et al. Active and adaptive plasticity in a changing climate. Trends Plant Sci. 2022;27(7):717–28. doi: 10.1016/j.tplants.2022.02.004 35282996

[31] Martin GM, Frazier DT, Loaiza-Maya R, Huber F, Koop G, Maheu J. Bayesian Forecasting in the 21st Century: A Modern Review. arXiv. 2022. https://arxiv.org/abs/221203471

[32] Macholdt J, Styczen ME, Macdonald A, Piepho HP, Honermeier B. Long-term analysis from a cropping system perspective: yield stability, environmental adaptability, and production risk of winter barley. European Journal of Agronomy. 2020;117:126056.

[33] Malikouski RG, Ferreira FM, Chaves SF da S, Couto EG de O, Dias KO das G, Bhering LL. Recommendation of Tahiti acid lime cultivars through Bayesian probability models. PLoS One. 2024;19(3):e0299290. doi: 10.1371/journal.pone.0299290 38442106 PMC10914267

[34] Miranda IR, Dias KOG, Júnior JDP, Carneiro PCS, Carneiro JES, Carneiro VQ. Use of Bayesian probabilistic model approach in common bean varietal recommendation. Crop Science. 2024;64(6):3163–73.

[35] Costa-Neto G, Crespo-Herrera L, Fradgley N, Gardner K, Bentley AR, Dreisigacker S, et al. Envirome-wide associations enhance multi-year genome-based prediction of historical wheat breeding data. G3 (Bethesda). 2023;13(2):jkac313. doi: 10.1093/g3journal/jkac313 36454213 PMC9911085

[36] Bartholomé J, Prakash PT, Cobb JN. Genomic prediction: progress and perspectives for rice improvement. Genomic Prediction of Complex Traits: Methods and Protocols. 2022. 569–617. 10.1007/978-1-0716-2205-6_21 35451791

[37] Kang M. A rank-sum method for selecting high-yielding, stable corn genotypes. Cereal Research Communications. 1988;16(1/2):113–5.

[38] Annicchiarico P. Cultivar adaptation and recommendation from alfalfa trials in Northern Italy. Journal of Genetics and Breeding. 1992;46:269.

