# Supplementary material for: Strategic risk analysis for the selection of stable and high-potential maize genotypes in multi-environment trials
Source: PLoS One. 2025 Jun 6;20(6):e0325454. doi: 10.1371/journal.pone.0325454 (PMC12143502; doi:10.1371/journal.pone.0325454)
Supplement: S1 Table — (DOCX) [file pone.0325454.s001.docx]

| **S1 Table.** Basic information of the 38 tested maize hybrids. | | | |
| --- | --- | --- | --- |
| **No.** | **Hybrids** | **Parentage** | **Crosses between** |
| 1 | H1 | KLM91005/3-1-1-1-1-2-1-1×K47/221 | Temperate × Temperate |
| 2 | H2 | KLM91015/2-2-1-1-1-1-2-1×K47/221 | Temperate × Temperate |
| 3 | H3 | KLM91015/2-2-1-1-2-1-1-1×K47/221 | Temperate × Temperate |
| 4 | H4 | KLM91015/2-2-1-1-2-1-2-1×K47/221 | Temperate × Temperate |
| 5 | H5 | YS16B-158-66-2×B73 | Tropical × Temperate |
| 6 | H6 | KLM91015/2-2-1-1-2-3-3-1×K47/221 | Temperate × Temperate |
| 7 | H7 | JH13A-520-161/1×MO17 | Tropical × Temperate |
| 8 | H8 | KLM91005/3-1-2-2-1-1-3-1×K47/221 | Temperate × Temperate |
| 9 | H9 | KLM91001/2-2-2-1-2-1-1-1×K47/221 | Temperate × Temperate |
| 10 | H10 | KLM91005/3-1-2-2-2-3-2-1×K47/221 | Temperate × Temperate |
| 11 | H11 | KLM91015/2-2-1-1-1-2-1-1×K47/3 | Temperate × Temperate |
| 12 | H12 | 7- CHTSEY,2002/90/41-1×K1264/5-1 | Sub-tropical × Temperate |
| 13 | H13 | K18x2-CHTHIY,2002/90/77-1×K1264/5-1 | Sub-tropical × Temperate |
| 14 | H14 | KLM91001/2-2-4-1-1-2-1-1×K1263/1 | Temperate × Temperate |
| 15 | H15 | KLM8915/1-3-1-8-1-1-1-1×K18 | Temperate × Temperate |
| 16 | H16 | B73 ×MO17(SC704) | Temperate × Temperate |
