# Supplementary material for: Strategic risk analysis for the selection of stable and high-potential maize genotypes in multi-environment trials
Source: PLoS One. 2025 Jun 6;20(6):e0325454. doi: 10.1371/journal.pone.0325454 (PMC12143502; doi:10.1371/journal.pone.0325454)
Supplement: Table S2 — (DOCX) [file pone.0325454.s002.docx]

| Table S2. Description of the 14 environments with their respective latitudes, longitudes, altitudes (m), soil group, mean temperature, rainfall (mm), and climate condition in regard to the maize trial. | | | | | | | | |
| --- | --- | --- | --- | --- | --- | --- | --- | --- |
| **No.** | **Environment,**  **Year** | **Latitude** | **Longitude** | **Altitude (m)** | **Soil group** | **Temperature**  **(˚C)** | **Rainfall**  **(mm)** | **Climate condition** |
| E01 | Karaj,  2022 | 35.49N | 51.00E | 1321 | Clay Loam | 16.45 | 380.00 | Temperate |
| E02 | Kerman,  2022 | 28.45N | 56.36E | 1200 | Clay Loam | 22.67 | 82.00 | Tropical |
| E03 | Mashhad,  2022 | 32.59N | 36.19E | 995 | Sandy Loam | 14.37 | 173.00 | Temperate |
| E04 | Kermanshah,  2022 | 34.80N | 47.26E | 1380 | Silty Clay | 16.90 | 321.80 | Temperate |
| E05 | Jiroft,  2022 | 28.40N | 57.44E | 630 | Sandy Loam | 23.48 | 168.00 | Tropical |
| E06 | Moghan,  2022 | 39.41N | 47.32E | 73 | Clay Loam | 16.86 | 454.00 | Temperate |
| E07 | Shiraz,  2022 | 25.46N | 52.43E | 1604 | Clay Loam | 16.88 | 149.00 | Tropical |
| E08 | Dezful,  2022 | 32.75N | 48.20E | 150 | Silty Clay Loam | 25.54 | 631.00 | Subtropical |
| E09 | Karaj,  2023 | 35.49N | 51.00E | 1321 | Clay Loam | 16.76 | 268.00 | Temperate |
| E10 | Mashhad,  2023 | 32.59N | 36.19E | 995 | Sandy Loam | 13.89 | 181.00 | Temperate |
| E11 | Kermanshah, 2023 | 34.80N | 47.26E | 1380 | Silty Clay | 17.00 | 205.60 | Temperate |
| E12 | Moghan,  2023 | 39.41N | 47.32E | 73 | Clay Loam | 17.85 | 327.00 | Temperate |
| E13 | Shiraz,  2023 | 25.46N | 52.43E | 1604 | Clay Loam | 17.53 | 222.00 | Tropical |
| E14 | Dezful,  2023 | 32.75N | 48.20E | 150 | Silty Clay Loam | 26.71 | 316.00 | Subtropical |
